# Quality of care in peripheral venous catheterization: A scoping review

**DOI:** 10.1590/0034-7167-2022-0578

**Published:** 2023-12-04

**Authors:** Saulo Pereira da Costa, Rodrigo Euripedes da Silveira, Damiana Aparecida Trindade Monteiro, Divanice Contim, Silmara Elaine Malaguti Toffano

**Affiliations:** I Universidade Federal do Triângulo Mineiro. Uberaba, Minas Gerais, Brazil

**Keywords:** Catheterization, Peripheral, Surveys and Questionnaires, Health Evaluation, Quality of Health Care, Review, Cateterismo Periférico, Encuestas y Cuestionarios, Evaluación en Salud, Calidad de la Atención de Salud, Revisión, Cateterismo Periférico, Inquéritos e Questionários, Avaliação em Saúde, Qualidade Assistencial, Revisão

## Abstract

**Objective:**

To map the current status of parameters and tools to assess quality of care related to peripheral venous catheter use in adults.

**Methods:**

Scoping review, conducted in 2022 in the MEDLINE, LILACS, CINAHL and SCOPUS databases and with a publication time limit from 2013 to 2022.

**Results:**

The sample consisted of 15 articles, summarized in the following categories: Indication, documentation and registration, coverage assessment, connection, stabilization and signs and symptoms inherent to the catheter. The use of a complete instrument, with the domains observed in this review, may have a positive impact on a more effective and safe clinical practice.

**Conclusions:**

The present review mapped the evidence about the insertion and maintenance of peripheral venous catheters that can be improved with training of good practices and the quality of the team, regarding the use of tools, materials and instruments for the evaluation of care.

## RESUMO

**Objetivo:**

Mapear o estado atual dos parâmetros e dos instrumentos para avaliar a qualidade de assistência relacionada ao uso de cateter venoso periférico em adultos.

**Métodos:**

Revisão de escopo realizada em 2022, nas bases de dados MEDLINE, LILACS, CINAHL e SCOPUS e com limite temporal de publicação de 2013 a 2022.

**Resultados:**

A amostra foi composta por 15 artigos, sintetizados nas seguintes categorias: indicação, documentação e registro, avaliação da cobertura, conexão, estabilização e sinais e sintomas inerentes ao cateter. A utilização de um instrumento completo, com os domínios observados nesta revisão, poderá impactar, de forma positiva, para uma prática clínica mais eficaz e segura.

**Conclusões:**

A presente revisão mapeou as evidências acerca da inserção e manutenção do cateter venoso periférico que podem ser aprimoradas com treinamento de boas práticas e qualidade da equipe, no que tange à utilização de ferramentas, materiais e instrumentos de avaliação do cuidado.

## RESUMEN

**Objetivo:**

Mapear el estado actual de los parámetros y herramientas para evaluar la calidad de la atención relacionada con el uso de catéteres venosos periféricos en adultos.

**Métodos:**

Revisión sistemática exploratoria, realizada en 2022 en las bases de datos MEDLINE, LILACS, CINAHL y SCOPUS y con un límite temporal de publicación de 2013 a 2022.

**Resultados:**

La muestra fue de 15 artículos, resumidos en las siguientes categorías: Indicación, documentación y registro, evaluación de la cobertura, conexión, estabilización y signos y síntomas inherentes al catéter. El uso de un instrumento completo, con los dominios observados en esta revisión, puede tener un impacto positivo en una práctica clínica más eficaz y segura.

**Conclusiones:**

Esta revisión ha mapeado las evidencias sobre la inserción y el mantenimiento de catéteres venosos periféricos que pueden mejorarse con una buena formación práctica y calidad del personal, en relación con el uso de herramientas, materiales e instrumentos para evaluar la atención.

## INTRODUCTION

In a general context, guidelines and recommendations for good practices in the prevention and control of healthcare-associated infections (HAIs) are standard in all health institutions. These guidelines guide care and are based on evidence, updated by government agencies and institutions, at national and international level^(1)^. In this context, peripheral venous catheters (PVC) are a consolidated therapeutic resource for carrying out intravenous therapy^(2)^, considered as the most used invasive clinical procedure in hospital environments^(3)^. It is estimated that more than 80% of patients admitted to hospital institutions require this procedure^(4)^, and in the United States of America, more than 300 million of these catheters are inserted in hospitalized patients annually^(3)^.

The adoption of good infection prevention practices in this process is contemplated by qualified and trained professionals, using aseptic technique and adequate sterile material. In addition, the infusion of solutions and drugs in recommended quantities and concentrations and the correct identification and maintenance of venous access are essential steps for patient safety, also preventing exposure of professionals to avoidable risks^(5)^. However, technical failures in the procedure of implantation of the PVCs are more frequent than is accounted for and are commonly incorporated into practice without proper discussion of the risks to which patients are exposed^(2,5)^.

In this context, these failures can lead to adverse events, often associated with the use of PVC^(6)^. Bloodstream infection (BSI) related to vascular catheterization is one of the most serious infections^(7)^. Prevalent factors related to phlebitis, infiltration, hematoma, thrombosis and thrombophlebitis are highlighted, with a mortality rate that can reach 40%^(6-7)^.

In view of the problem exposed, the need for standardized instruments and mechanisms to evaluate the quality of care related to the use of PVCs is reinforced. Thus, measures can be directed to prevent infections and local complications, as well as to implement strategies for professional qualification singularized to the adoption of the best practices of insertion and maintenance of the vascular catheter^(7-9)^. Although there are assessment tools related to the use of peripheral venous catheters, mainly related to phlebitis, there is a shortage of effective tools and measures that assess the quality of care^(10-11)^.

In 2018, specialists in this subject developed the Peripheral Intravenous Catheter Mini Questionnaire (PIVQ-miniQ)^(12)^. The PIVQ-miniQ instrument consists of 16 items with dichotomous yes and no answers, subdivided into four domains, evaluating the PVC insertion site, dressing and connections, clinical indication and documentation. The score of the instrument varies from a score range between zero and 16 points, and the lower the score, the lower the prevalence of problems affecting the quality of care^(11)^.

This study is relevant to obtain elements that can support strategies related to the quality of care relate to the use of PVCs and to expand knowledge about existing instruments used for this purpose in the literature.

## OBJECTIVE

To map the current status on parameters and tools to assess quality of care related to peripheral venous catheter use in adults.

## METHOD

### Ethical aspects

Because this is a scoping review, using data in the public domain, the study was not submitted to the research ethics committee.

### Study type

This is a descriptive and qualitative study, outlined as a scoping review, following the review method proposed by the Joanna Briggs Institute (JBI)^(13)^ version 2020 and the recommendations of the Preferred Reporting Items for Systematic Reviews and Meta Analyses extension for Scoping Reviews (PRISMA-ScR)^(14)^. It is noteworthy that the scoping review results in a new approach of systematized review of the scientific literature, with growth in national and international publications in the last ten years. The method allows examining evidence, and identifying existing gaps and the key concepts in a defined thematic area^(13)^.

### Methodological procedure

In the present study, five steps were listed: formulation of the guiding question (“What are the parameters that assess the quality of care related to PVC in adults in the in-hospital environment?”); search of relevant literature databases on this topic and data extraction; search and analysis of studies and presentation and synthesis of results. For the construction of the study question, the Population, Concept, Context PCC^(13)^. The acronym corresponded, respectively, to P - population: adults who are using a PVC; C - concept: instruments/questionnaires and parameters that assess the quality of care related to PVC in clinical practice; Context: Hospital units that use PVCs.

It is important to highlight that in this study the concept of PVC relates to the use of a catheter inserted into the peripheral vein that is combined with accessories such as extenders and cannulas. In addition, there are also devices used for stabilization and coverage of these PVCs.

### Data collection and organization

The search was conducted between February and April 2022 with the keywords: “adult”, “peripheral venous catheterization”, “health assessment” and “quality of health care”, in the databases Medical Literature Analysis and Retrieval System Online (MEDLINE), through its free PubMed interface; Latin American and Caribbean Literature in Health Sciences (LILACS), through the Virtual Health Library (VHL); Cumulative Index To Nursing and Allied Health Literature (CINAHL) and SciVerse Scopus (SCOPUS). Descriptors appropriate to the databases studied were chosen (Health Sciences Descriptors - DeCS and Medical Subject Headings - MeSH).

Chart 1 shows the strategies elaborated with the descriptors used with the help of the Boolean operators AND and OR to compose the search, in addition to quantifying the articles located and selected in each database.

**Chart 1:** Strategy for searching the databases for articles, 2022

SOURCE	SYNTAX
BVS/LILACS	(“Avaliação em Enfermagem” OR “Nursing Assessment” OR “Evaluación em Enfermería” OR “Avaliação em Saúde” OR “Health Evaluation” OR “Evaluación en Salud”) AND (“Cateterismo Periférico” OR “Cateterismo Venoso Periférico” OR “Catheterization, Peripheral” OR “Peripheral intravenous cateter”) AND “Adult” OR “aged”
SCOPUS	(TITLE-ABS-KEY ((nursing AND assessment) OR (health AND evaluation)) AND TITLE-ABS-KEY ((catheterization, AND peripheral) OR (peripheral AND intravenous AND cateter)) AND TITLE-ABS-KEY ((adult)) AND TITLE-ABS-KEY ((quality) OR (health AND care)))
CINAHL	((nursing assessment OR health evaluation)) AND ((catheterization, peripheral OR peripheral intravenous cateter)) AND (adult OR aged ))
MEDLINE via PubMed	((Nursing Assessment OR Health Evaluation)) AND (Catheterization, Peripheral OR Peripheral intravenous cateter) AND (Quality of Health Care OR Quality OR Health Care) AND (adult OR aged)

As inclusion criteria we considered: primary articles and systematized literature reviews (systematic review, integrative and scoping reviews) that corresponded to the study objects, published between 2013 and 2022, available in full and with no limit as to the published language. This time frame was based on the intensification of publications and discussions about the management of quality of care in relation to the use of PVC, as well as the concept of difficult peripheral venous puncture (DPVP). It is worth mentioning that situations of DPVP have always existed in nursing practice, but with advances in knowledge, these professionals have adopted protocols for the management of peripheral venous puncture (PVP), avoiding multiple punctures and other possible complications, which has a direct impact on the quality of care related to the use of PVCs.

The exclusion criteria were: duplicate studies, theses, dissertations, non-systematized literature reviews, letters to the editor, opinion articles, annals, booklets and advance note articles. In the subsequent stage, the included studies were read in full, with critical evaluation and interpretation of the results with knowledge synthesis. For this purpose, the content analysis method was used, which allows, by means of a critical and analytical description, to classify the components of the meaning of the messages obtained in the articles into different categories, resulting from the grouping of classes of elements that gather characteristics in common^(15)^.

### Data analysis

In the screening phase, the selection of articles had the duplicate studies removed. Two independent researchers performed a careful reading of titles and abstracts, basing the selection on the aforementioned eligibility criteria; when there was no consensus, the evaluation of a third reviewer was used. The final stages of information extraction and delimitation also occurred by two independent reviewers, using a form developed by the investigators to characterize the study, mapping author, title, year and country of publication, study design and sample, objective or interventions performed, outcomes found and level of evidence of the study.

The classification regarding the level of evidence took into account the classification system recommended by the JBI^(16)^, which is comprised of five levels of evidence, being level 5 (expert opinion), level 4 (descriptive observational studies such as cross-sectional studies for example), level 3 (analytical observational studies such as cohort studies and case control for example), level 2 (quasi-experimental studies), and level 1 (experimental studies including systematic review and randomized clinical trial). After this stage, the articles were characterized, with synthesis and description of the results related to the study question, being grouped into guiding axes that characterize factors determining quality of care related to PVCs.

## RESULTS

**Figure 1:**
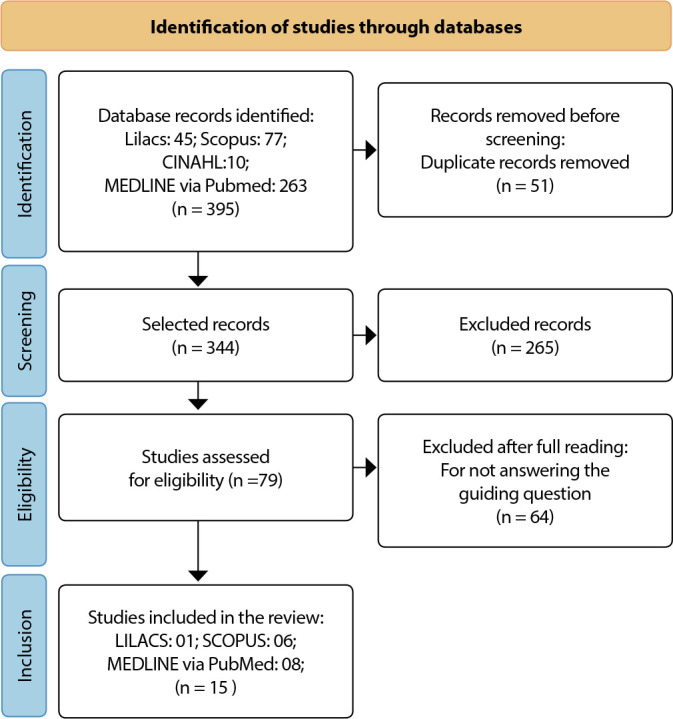
Flowchart for the selection of scoping review studies (PRISMA-ScR)

The search identified 395 studies, of which 51 were removed due to duplication. The remaining 344 studies were selected by title and abstract, of which 79 were included for eligibility assessment, with full text reading by two independent reviewers. Then, after this reading, another 64 studies were removed for not answering the guiding question of the study, leaving 15 studies that made up the final sample. The selection of articles was presented in the PRISMA Flowchart for Scoping Reviews (PRISMA-ScR), Figure 1.

The studies included in the review are presented in Chart 2, according to author, title, year and country of publication, study design, objective or interventions performed, outcomes found and level of evidence of the study. The studies were published between 2015 and 2022, with the highest number of publications in 2020, (27%), and overall presented a strong level of evidence with seven studies (47%) classified as Level 1. Regarding the country of origin, 13 (86%) studies were conducted in Australia. This can be justified by the large number of collaborating researchers belonging to the Australian research group, called Alliance for Vascular Access Teaching and Research (AVATAR), which in partnership with researchers from all over the world, including Brazilian researchers, has stood out in advancing knowledge in vascular access, including PVCs. Different research methods are being developed, such as clinical trials, systematic reviews and knowledge translation studies related to vascular access devices^(17-18)^. In other words, the AVATAR group carries out scientific work committed to the evolution of health services, seeking to eliminate ineffective practices and replace them with innovative solutions, ensuring better patient care and directly impacting the economic issue of these services^(19)^.

**Chart 2:** Studies analyzed according to Database/Journal, title, authors, methodological design, year of publication, country of origin of the study, 2022

Author/Title	Year Country	Design/Sample	Objective/ Interventions	Outcomes	Level of Evidence
Carr, Rippey, Cooke^(20)^ “Factors associated with peripheral intravenous cannulation first time insertion success in the emergency department”.	2019 Australia	Prospective observational 879 patients (n=1,201 PVC)	To identify the incidence of factors associated with peripheral intravenous infusion and the success rate of first versus two or more punctures.	Incidence of first puncture success rate = 645/879. Success related to age and palpability of the patient’s vein, as well as practitioner’s confidence and experience.	3.e
Sweeny et al.^(21)^ “The experience of patients at high risk of difficult peripheral intravenous cannulation: An Australian prospective observational study”.	2022 Australia	Prospective observational n=1,084	To identify patients with difficult peripheral intravenous cannulation (DPIVC)	Patient characteristics (absence of visible or palpable vein, history of difficult PVC) type of PVC (larger caliber), site (hand and wrist) were pointed out as factors.	3.e
Marsh et al.^(22)^ “Peripheral intravenous catheter non-infectious complications in adults: A systematic review and meta-analysis”.	2020 Australia	Systematic review with 103 studies (n= 96,777 PVC)	To point out main peripheral complications related to PVC use and where they were inserted.	Incidence of phlebitis (23.8%) and infiltration (13.7%) being significantly higher when catheters were inserted in the emergency department.	1.b
Mihala et al.^(23)^ “Phlebitis signs and symptoms with peripheral intravenous catheters”.	2018 Australia	Descriptive observational n=3,283	To calculate incidence of signs and symptoms for diagnosis of phlebitis and their correlations.	Considerably low incidence and only correlations observed were heat x stiffness, heat x swelling and heat x erythema.	4.a
Marsh et al.^(24)^ “Devices and dressings to secure peripheral venous catheters: A Cochrane systematic review and meta-analysis”.	2017 Australia	Systematic review with meta-analysis of 6 studies (n=1,539)	To evaluate the effects of fixation devices on the incidence of catheter loss.	Less PVC detachment with transparent dressing compared to gauze.	1.a
Schmutz et al.^(25)^ “Dislodgement forces and cost effectiveness of dressings and securement for peripheral intravenous catheters: A randomized controlled trial”.	2020 Germany	Randomized controlled trial n= 209	Force required to remove a PVC versus four dressing and fixation methods.	Sterile absorbent dressing covered by two incisive polyester wool elastics had higher strength and better cost-effectiveness compared to the other techniques.	1.c
Rickard et al.^(26)^ “Dressings and securements for the prevention of peripheral intravenous catheter failure in adults (SAVE):a pragmatic, randomized controlled, trial”.	2018 Australia	Randomized controlled trial n= 1,807	Effectiveness and costs of three types of standard borderless polyurethane dressing.	No significant results were observed. These methods are associated with loss of PVCs and low durability.	1.c
Corley et al.^(27)^ “Peripheral intravenous catheter securement: An integrative review of contemporary literature around medical adhesive tapes and supplementary securement products”.	2022 Australia	Integrative review with 19 studies n=43,683 PVC	Synthesize the evidence related to medical adhesive tapes for PVCs.	The quality assessment identified high risk of bias or confounding factors. The authors concluded that the evidence is limited.	4.b
Keogh et al.^(28)^ “Implementation and evaluation of short peripheral intravenous catheter flushing guidelines: a stepped wedge cluster randomized trial”.	2020 Australia	Randomized controlled trial n= 619	Evaluated the impact of a multifaceted intervention for PVC maintenance.	lushing with 0.9% NaCL administered via an already prepared syringe with ready-to-use system showed a difference in risk (-8%, 95% CI -14 to -1, p = 0.032) compared to the control group with standard care.	1.d
Marsh et al.^(29)^ “Securement methods for peripheral venous catheters to prevent failure: A randomized controlled pilot trial”.	2015 Australia	Randomized controlled trial n=89	Evaluating the effectiveness of four safety methods to prevent PVC-related failures	Catheter failure was lowest in the tissue adhesive group (14%) and highest in the control group (38%).	1.c
Marsh et al.^(30)^ “How many audits do you really need?: Learnings from 5-years of peripheral intravenous catheter audits”.	2021 Australia	Prospective observational n=2,274 PVC	Determine the optimal number of PVC patients for clinical audits.	Authors defined that optimal values should be between 100 and 250 PVC per audit round, depending on the prevalence of complication.	3.e
Dutra et al.^(31)^ “Prevention of events with vascular catheters: Validation of an instrument”.	2021 Brazil	Methodological study n=50	To validate an instrument that identifies factors that hinder PVC insertion and maintenance.	Instrument proved to be valid (high Content Index) and reliable (through Interobserver reliability using Kappa coefficient).	4.c
Yagnik, Graves, Thong^(32)^. “Plastic in patient study: Prospective audit of adherence to peripheral intravenous cannula monitoring and documentation guidelines, with the aim of reducing future rates of intravenous cannula-related complications”.	2017 Australia	Prospective observational n=102	To improve compliance of PVC documentation and monitoring through three “The plastic in patient - PIP” interventions.	Documentation improved in the post-intervention group (36.4 x 50%, p = 0.025). Early identification for non-indication of PVC and a trend towards a reduction in PVC-related phlebitis also had positive results post-intervention.	3.c
Webster et al.^(33)^ “Clinically indicated replacement versus routine replacement of peripheral venous catheters”.	2019 Australia	Systematic review with meta-analysis of 9 studies (n = 7,412)	To evaluate the effects of PVC removal when clinically indicated versus routine catheter removal.	There is no clear difference in the rates of catheter-related infection, phlebitis and pain in the two groups. There is moderate-certainty evidence that catheter infiltration and blockage are lower when access is changed routinely. With moderate certainty evidence, there was a reduction in device-related costs when clinically indicated.	1.a
Ray-Barruel et al.^(34)^ “The I-DECIDED clinical decision-making tool for peripheral intravenous catheter assessment and safe removal: a clinimetric evaluation”.	2020 Australia	Methodological study n=68	To validate an 8-step tool for device assessment and decision making.	“I-DECIDED” tool demonstrated strong content validity, with high reliability and replicability for advising PVC-related decision making and assistance.	4.c

*PVC - Peripheral venous catheter; PVP - Peripheral venous puncture.*

Regarding the design, the majority, 12 (80%) studies derived from these three types of research: Prospective Observational, Randomized Controlled Study and Review Articles, containing four studies in each of these methodologies applied.

## DISCUSSION

This study contributed to investigations on which issues directly impact on the quality of care related to PVC in adults. The results obtained demonstrate the scarcity of instruments that can contribute to evaluate this issue. The categories described below were created by combining studies by thematic approach, which dealt with quality of care with a predominant focus on the following themes.

### Signs and symptoms related to the insertion site of the peripheral venous catheter

Among the studies analyzed, the lack of an adequate instrument that evaluates and supports robust clinical decision making to guide health professionals who perform peripheral venipuncture in adults was explicit^(20-21)^. A study identifying the incidence and factors associated with successful PVC insertion at the first puncture attempt in Western Australian emergency departments used logistic regression modeling to evaluate 1201 PVCs inserted in 879 patients. The authors identified that the first puncture success rate was 73%, with 128 (15%) requiring a second attempt and 83 (9%) requiring three or more attempts^(20)^.

The success of the first attempt may be related to several factors, such as patient age, palpation of the vein to be punctured in addition to clinical factors, and also related to the health professional who performs the puncture, with statistically significant results being found when performed by health professionals with greater confidence and greater insertion experience^(20)^.

Regarding signs and symptoms, the inherent failures of peripheral venous catheterization have been described as painful and uncomfortable, and repeated punctures substantially increase the risk of infection^(21)^. A meta-analysis, which totaled approximately 80,000 catheters, pointed out the main peripheral complications related to the use of PVC. Among them, phlebitis (23.8%), infiltration (13.7%), occlusion (8%), leakage (7.3%) and pain (6.4%) were the most frequent. This same study pointed out that infiltration had a significantly higher prevalence in the case of catheters inserted by the emergency department than those inserted in other clinics^(22)^. These results provide nurses with a strong evidence base for the development of effective interventions for the prevention of PVC-related adverse events.

Another study, conducted in three large general hospitals in Australia, calculated the incidence of eight signs and symptoms used for the diagnosis of PVC-related phlebitis and the level of correlation between them. This included more than 20,000 records of daily observations of six signs (swelling, erythema, leakage, palpable vein, purulent discharge and warmth) and two symptoms (pain and stiffness) at 5,907 catheter insertion sites. Most signs and symptoms of phlebitis occurred only occasionally, and vein stiffening had the highest incidence (5.7%). The study found that the incidence of signs and symptoms of phlebitis was considerably low and the only correlations observed between each other were heat with stiffness, heat with swelling, and heat with erythema^(23)^.

### Conditions of catheter stability, coverage and connections

In the meta-analysis conducted with reports from relevant randomized controlled trials (RCTs), data from 1,539 PVCs were analysed to evaluate the effects of dressings and fixation devices on the incidence of catheter loss. The RCTs made four comparisons, namely: transparent dressings versus gauze; transparent dressings with a border versus a fixation device; transparent dressings with a border versus tape; and transparent dressing versus adhesive plaster^(24)^.

The results indicate that fewer catheter dislodgements or accidental removals occur with transparent dressings compared with gauze. It is known, however, that the relative effects of transparent dressings and gauze on phlebitis and infiltration are unclear, and for this reason the authors conclude that it cannot be stated whether any fixation device is better than another in PVC fixation, which is also proven in other studies^(25-27)^. It is important to emphasize the consideration of the cost-effectiveness of the fixation device, since health services may have only adhesive plaster for fixation and no access to transparent dressings, for instance.

A study conducted in Germany compared the force required to dislodge a PVC with four commonly used dressing and fixation methods. The accesses of 209 volunteers were observed, with similar tension applied between them, pulling until PVC loss occurred. The greatest resistance against the force applied to remove the access could be observed with a sterile absorbent dressing covered by two incisive polyester wool elastics. This RCT also demonstrated that this type of dressing is more cost-effective than other techniques and is strongly indicated against accidental removal^(25)^.

Cost should always be a consideration when it comes to PVC stability, as current methods of dressing and fixation are commonly associated with PVC loss and poor durability, requiring the simultaneous use of multiple products^(24-26)^. Cost is a determining factor in product choice. Innovations to achieve effective and more durable dressings and holds, and the use of more sensitive instruments and RCT to evaluate their efficacy, are needed^(25)^.

The catheter connection device was evaluated in another controlled trial, to assess the impact of a multifaceted intervention for maintaining short PVC, comparing commonly standard practice (venipuncture with PVC and flushing with 0.9% sodium chloride, administered via a professionally prepared syringe) with a technique-specific intervention (venipuncture with PVC and flushing with 0.9% sodium chloride, administered via an industry-prepared syringe, with a ready-to-use system). PVC failure had a significantly lower occurrence in the intervention group ^(27)^. These results reinforce the need for evidence that can improve PVC-related quality of care.

New technologies emerge as safety methods to prevent failures related to peripheral venous catheterization. A controlled trial conducted in Australia randomly allocated groups to standard polyurethane dressing (control), tissue adhesive, polyurethane dressing with edge, or sutureless fixation device. The primary end point was PVC loss, defined as premature removal of the device before the end of therapy because of pain, blockage, leakage, accidental removal, or local or catheter-related bloodstream infection^(28)^.

The results showed that PVCs on average remained viable for 2.6 days in all study groups. Catheter failure was lowest in the tissue adhesive group (14%) and highest in the control group (38%). No patient had local or catheter-related infection. The authors infer that the current standard polyurethane dressing alone does not prevent many cases of access failure or loss, while tissue adhesives appear promising although not suitable for all patients^(29)^.

### Documented records of insertion and maintenance

Regarding the studies of reports and clinical records intricate with PVC processes, the results of two audits registered in a hospital and a large clinic were found. It is also emphasized that the audit of PVC care translates into an effective method to promote infection prevention and improve the quality of care.

When studying a cross-sectional dataset of clinical audits collected over five years (2015 to 2019) in a large Australian metropolitan hospital across a wide range of clinics, 2274 PVCs were reviewed. The aim was to determine the optimal number of PVC patients for clinical audits for evidence-based surveillance. The result showed that 475 cases (21%) had some complication. Accuracy was not significantly improved by audits with more than 150 patients with a complication rate of 20%, nor in those performed with more than 200 patients with a complication rate of 50%, suggesting that the ideal values should be between 100 and 250 PVCs per audit round, depending on the prevalence of complication^(30)^.

Ideally, each PVC should be audited, but this is rarely feasible. In this sense, in order to ensure that hospitals capture efficient and timely data that also reasonably reflect the quality of care; the use of specific and comprehensive questionnaires is fully encouraged, supporting evidence-based decision making, while also offering resources for health administrators and policymakers.

A study conducted in Brazil validated an audit tool for insertion and maintenance of vascular catheters. It was observed whether the coverage and devices/connections, in addition to being correctly identified, were also within the established expiration date. Authors also highlight the importance of this type of instrument to identify weaknesses in the service, train teams, review work processes and improve quality of health care and patient safety^(31)^.

In another study also conducted through audit, the aim was to improve documentation compliance and monitor PVC-related lines of care in the medical ward of a secondary care center following interventions. The ‘Plastic in Patient’ (PIP) section was applied as a dedicated column in the evolution chart, identifying inpatients with PVC requesting evaluation of the indication at daily multidisciplinary meetings. Results demonstrated that documentation improved significantly in the post-intervention group. Similarly, early identification of non-indicated PVCs improved in the post-intervention group and a trend towards a lower rate of phlebitis was also observed^(32)^.

In fact, the authors observed that the inclusion of new forms to be filled in the daily routine of health care was reflected in considerable results for better control of actions in the clinic related to venipunctures, as well as suggesting a tendency to reduce phlebitis rates, corroborating the perspective of expanding the research instruments, as simple and economical interventions that result in improvements in adherence to the guidelines of professional health practice^(32)^.

### Indication of peripheral venous catheter use

In the last category presented in this scoping review, two studies focused on the need for a prescription and the information required to indicate the use or removal of PVC in an objective manner.

One study evaluated the effects of PVC removal when clinically indicated compared with routine catheter removal and repositioning. Studies were searched in different databases for RCTs in which the population consisted of hospitalized patients receiving continuous or intermittent infusions via PVCs. The final trial included nine studies with 7,412 participants. All trials reported an incidence of thrombophlebitis, although there was no clear difference in the prevalence of this adverse event when catheters were changed following clinical indication or routinely^(33)^.

The authors concluded that there was no clear difference in the rates of catheter-related infection or any type of bloodstream infection, thrombophlebitis, morbidity, mortality and pain related to clinically indicated or routine PVC replacement. There is moderate certainty evidence that catheter infiltration and blockage are likely to be lower when access is routinely changed. Finally, there is moderate certainty evidence that clinically indicated catheter replacement or removal reduces device-related costs. The suggested guidance is that the gold standard for this procedure is catheter replacement/exchange when there is a clinical indication, for example in the presence of signs of infection, obstruction or infiltration^(33)^.

Another study conducted aimed to describe the clinimetric validation of the ‘I-DECIDED’ assessment and decision-making tool for PVC use. It is an eight-step tool derived from international guidelines for vascular access, structured in the form of a mnemonic process for device evaluation and decision-making; whose clinimetric evaluation process was conducted in three different phases^(34)^.

The results showed that the ‘I-DECIDED’ tool demonstrated strong content validity among independent international experts, with high reliability and acceptance by the multiprofessional team in the testing phase. The authors conclude that the tool is easy to understand and replicable for advising PVC-related decision making and care. They recommend further studies to evaluate the outcome of the use of this tool in clinical practice in other locations^(34)^.

### Limitations of the Study

There is a scarcity of studies on this theme and with robust and replicable methodology among Latin American countries, in contrast to the large number of studies found in Australia, which suggests that the inclusion of other databases, associated with the increase in the period to be studied, may produce more comprehensive data for assessment.

### Contributions to the nursing Field

The scoping review approach allowed the identification of parameters and instruments that evaluate the quality of care related to peripheral venous catheters. Thus, these results may broaden evidence-based knowledge and strengthen the preventive measures to control PVC-related failures in the adult population, as well as encourage further studies on this topic.

## CONCLUSION

The present review mapped the evidence that pointed out that PVC insertion and maintenance can be improved with the training of good practices and the quality of the team, regarding the use of tools, materials and instruments for the assessment of care. The need for frequent observation of the catheter insertion site, identification and indication of the catheter was found to be important. In addition, the need for patient guidance and family integration regarding care for venous access maintenance is emphasized.

The use of a single instrument that can include all the facets addressed in the study is recommended for more effective and safe care. In this direction, efforts are suggested to address the problem associated with the complications of PVC use, which in addition to causing trauma and discomfort to patients, require longer hospitalization with increased costs related to health care.
